# An Integrated Strategy for Implementation of Dried Blood Spots in Clinical Development Programs

**DOI:** 10.1208/s12248-015-9860-3

**Published:** 2016-02-08

**Authors:** Prajakti A. Kothare, Kevin P. Bateman, Marissa Dockendorf, Julie Stone, Yang Xu, Eric Woolf, Lisa A. Shipley

**Affiliations:** Pharmacokinetics, Pharmacodynamics and Drug Metabolism, Merck & Co., Inc., 770 Sumneytown Pike, West Point, Pennsylvania 19486 USA

**Keywords:** bridging, dried blood spots, MK-8931, population PK

## Abstract

Dried blood spot (DBS) sample collection has gained increased interest across the pharmaceutical industry as a potential alternative to plasma for pharmacokinetic (PK) evaluations. However, regulatory guidelines and examples of late-stage clinical trial applications in the literature are lacking. This paper communicates Merck’s strategy for the implementation of DBS exemplified by experience on a late-stage program (MK-8931). In this program, DBS was proposed as the sole matrix for phase 3 studies to decrease logistical burden in an aging target patient population (Alzheimer’s disease). *In vitro* and bioanalytical tests demonstrated initial method feasibility and suitability for further evaluations in the clinic. An *in vivo* dataset was developed initially in healthy subjects (phase 1 study) and then in patients (phase 2/3 study) to establish a quantitative relationship between the blood and plasma concentrations (bridging dataset) using descriptive and population PK analyses. This allowed for PK conclusions to be seamlessly drawn across the clinical program without impact from the choice of matrix. This integrated information package (*in vitro*, bioanalytical and clinical) was presented to major regulatory agencies (FDA and EMA) for regulatory input. Based on this package, regulatory concurrence was gained on accepting DBS as the sole matrix in late-stage clinical trials.

## INTRODUCTION

Since its original application for the detection of phenylketonuria in neonates half a century ago, dried blood spots (DBS) have gained popularity as a screening tool for various diagnostic tests including metabolic disorders, therapeutic drug monitoring, and HIV infection in neonates (1–3). The pharmaceutical industry and regulators have continued to explore the potential of DBS as a viable alternate matrix for pharmacokinetic analyses (4–6). DBS was initially evaluated at Merck in 2001 for discovery stage PK studies and subsequently implemented for the pediatric development program of an anti-HIV compound in 2009. Since then, a number of compounds have been evaluated for initial *in vitro* and bioanalytical feasibility to implement DBS. A subset of these has progressed to implementation of DBS in clinical trials, including late-stage clinical trials. Strategically, Merck has chosen to primarily focus the application of DBS towards late-stage patient studies (phase 2 and/or 3) where it has the potential to render greater impact. In our opinion, the value proposition for DBS from a clinical perspective is as follows:

- To add flexibility in the collection of PK data in phases 2 and 3 studies: Typically, sparse PK samples in phases 2 and 3 studies are constrained to limited time windows during a clinic visit. DBS, particularly in an out-patient setting, expands the window for access to such data. This may be especially beneficial for drugs with long half-lives or long acting formulations to evaluate steady-state or time for washout, or where clinical endpoints are collected by patient-completed diaries or are episodic (*e.g*., migraine, asthma, or erectile dysfunction trials). However, sampling methods in an out-patient setting need to mature further to gain adequate precision for pharmacokinetic modeling.
- Decreased patient burden (blood volume) in vulnerable populations: The smaller sample volumes typically associated with DBS (three spots of ∼20–40 μL each) *vs*. typical plasma samples (∼200–1000 μL) are clinically attractive for vulnerable populations where blood volumes are a clinical or ethical concern (*e.g*., younger pediatric or elderly populations). However, DBS should not be considered an automatic choice for studies in such populations. Equal consideration should be given to liquid microsampling approaches.
- Improved logistical feasibility: DBS sampling offers a number of logistical advantages (*e.g*., ambient temperature storage or shipping, no need for specialized equipment such as refrigerated centrifuges and simplified sample preparation) that may reduce operational burden associated with PK sampling in larger multi-center patient trials and lead to potential cost savings. A reduced operational burden may encourage greater participation of clinical sites for PK evaluation in phases 2 or 3 trials, and thereby enrich the database for characterization of the population pharmacokinetics and exposure-response relationships. Prior to implementing ambient shipping for DBS, the extended stability of DBS samples should be established to ensure integrity of the samples.

DBS has been used extensively for the measurement of endogenous biomarkers of disease (7,8). Merck is exploring the use of DBS for clinical laboratory tests and biomarkers. Continued development of these approaches and coordination with PK measurements is anticipated to further enhance the value proposition for DBS in clinical trials.

Despite the upside potential of DBS, companies and regulators have wrestled with its utility and regulatory expectations compared to traditional matrices. Recently, the European Bioanalysis Forum (EBF) and IQ have published position papers reflecting a range of opinions on DBS (9,10). However, industry regulatory consensus positions and regulatory guidelines for DBS in clinical development programs have been slow to emerge. Further, with few exceptions, the literature has focused on technological challenges and bioanalytical considerations. Rowland and Shepard provide an overview of the requirements for the interpretation of DBS data in development and addressed some of the regulatory considerations that would pave the way to gaining acceptance for DBS (11). A theoretical assessment of pharmacokinetic considerations in the interpretation of DBS data has been published by Emmons and Rowland (12). However, the literature lacks in-depth guidance on pharmacokinetic analyses, modeling, or late-stage clinical considerations, especially when integrating across a clinical program with studies that include both plasma and DBS sampling.

The use of DBS should be thoughtfully weighed on a case-by-case basis with consideration to pros/cons of its use relative to traditional matrices for a given program. Successful implementation hinges on prospective multi-disciplinary (*e.g*., Bioanalytical, pharmacokinetic-pharmacodynamic (PKPD), Clinical Pharmacology, regulatory) planning. As most clinical development programs employ plasma sampling for phase 1 studies, a robust *in vivo* bridging strategy should be developed that allows quantitative inter-conversion of pharmacokinetic information between matrices. This allows pharmacokinetic conclusions to be drawn across studies agnostic to matrix. The overarching PKPD objectives for the program (*e.g*., evaluation of intrinsic/extrinsic factors, exposure-response) should remain a core consideration and not be impacted by the choice of the bioanalytical matrix.

**Fig. 1:**
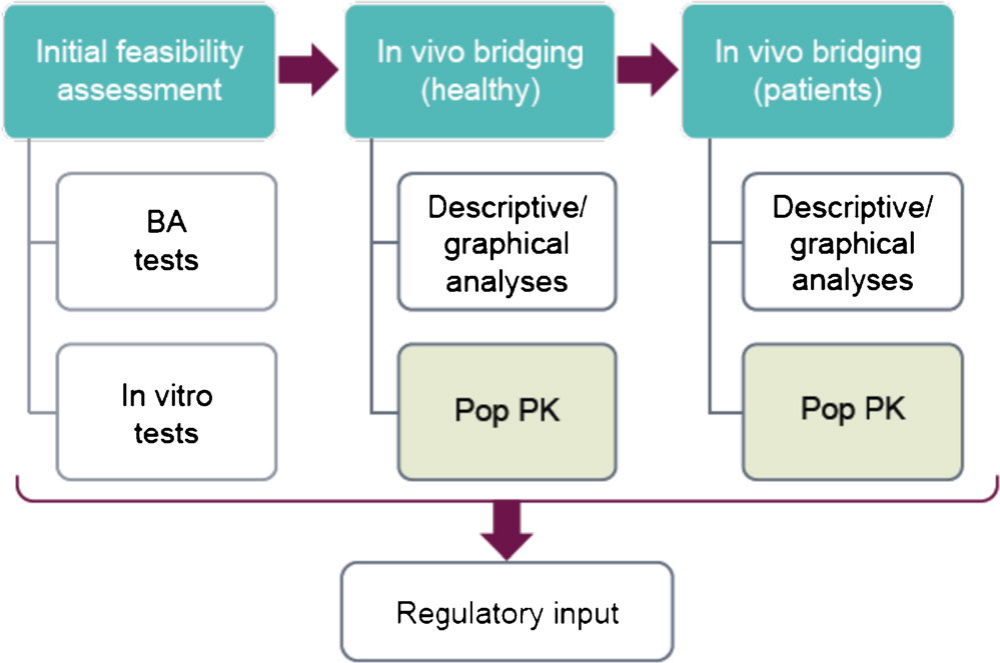
Components of the integrated DBS strategy

This paper presents Merck’s strategy for the application of DBS in clinical programs which has been shaped by experience gained from implementation on clinical programs. This augments an earlier company position paper where initial experience, bioanalytical, and logistical considerations were communicated (6). Of note, this summary is intended to inform the scientific community of an emerging area of interest with limited industry or regulatory precedence and is not intended to represent a broader consensus position by regulators or industry. The strategy is composed of staged implementation steps where initial methodological feasibility is established through a series of *in vitro* and bioanalytical evaluations. DBS is introduced in a staged manner in the clinic whereby DBS samples are taken concurrently with plasma initially in a healthy subject study and then in patients. Descriptive and population PK analyses of these data are conducted in a “learn and confirm” paradigm. Regulatory feedback is sought on this comprehensive data package (Fig. 1). Implementation of this strategy is exemplified through experience gained from MK-8931, a clinical program where regulatory concurrence was gained for the acceptance of DBS as the sole PK collection matrix for late phase trials.

## CASE STUDY: MK-8931

### Strategic Rationale

MK-8931 is currently in late-stage development for Alzheimer’s disease. DBS would allow for shipping at ambient temperature and render a number of other logistical advantages that could encourage clinical site participation and facilitate faster study enrollment for large multi-site clinical trials. Additionally, the reduced blood volume associated with DBS collection was anticipated to reduce patient burden. The goal of implementation was to pursue DBS as the sole matrix for late-stage clinical trials. The characterization of exposure-response relationships was considered an important element for dose justification. Thus, the bridging package needed to be robust to allow clinical studies to be pooled across the clinical program for population PK and exposure-response analyses in anticipation of regulatory submission and labeling.

### Initial Feasibility Assessment

Initially, a set of *in vitro* and bioanalytical assessments were conducted to ascertain methodological feasibility prior to further evaluations in the clinic. These tests were conducted over a clinically relevant concentration range and required good communication between the bioanalytical and PKPD scientists.

#### *In Vitro* Tests

As detailed in the publication of Rowland and Emmons (12), plasma protein binding, blood–cell to unbound plasma concentration ratio (*ρ*), and hematocrit are important *in vitro* determinants of the blood to plasma ratio and the suitability of DBS as a pharmacokinetic matrix. Emmons and Rowland hypothesize that compounds with a blood to plasma ratio ranging from 0.55 to 2, and non-concentration-dependent unbound fraction (fu) and *ρ* can be equally analyzed by blood or plasma (5,12).

The average MK-8931 *in vitro* blood to plasma ratio was 1.22 at 1 μM based on radiolabelled experiments. The blood to plasma ratio was concentration independent in the range of 0.03 to 10 μM (bracketing the clinical concentration range). MK-8931 was modestly plasma protein bound (~65% in human plasma) and not concentration dependent in the range of 0.03 to 1 μm. The *in vitro* tests showed that DBS or plasma would be equally valid as a matrix for MK-8931 PK assessments.

#### Bioanalytical Tests

Prior disseminations summarize Merck’s strategy for evaluating bioanalytical feasibility (6,13). These tests encompass assay sensitivity and range, DBS card type and extraction methods, the impact of hematocrit on the bioanalytical method over a wide range, impact of spot volume/homogeneity, stability (including relevant metabolites) at extremes of temperate and relative humidity (potentially encountered during shipping), and any considerations specific to the quantitation of the molecule (*e.g*., concentration-dependent binding to target and/or plasma). There are currently no established regulatory guidances for bioanalytical method development or validation specific to DBS. Hence, following a decision to pursue DBS, regulatory bioanalytical guidelines, internal SOPs, and industry best practices for plasma assays are applied for bioanalytical validation.

**Fig. 2:**
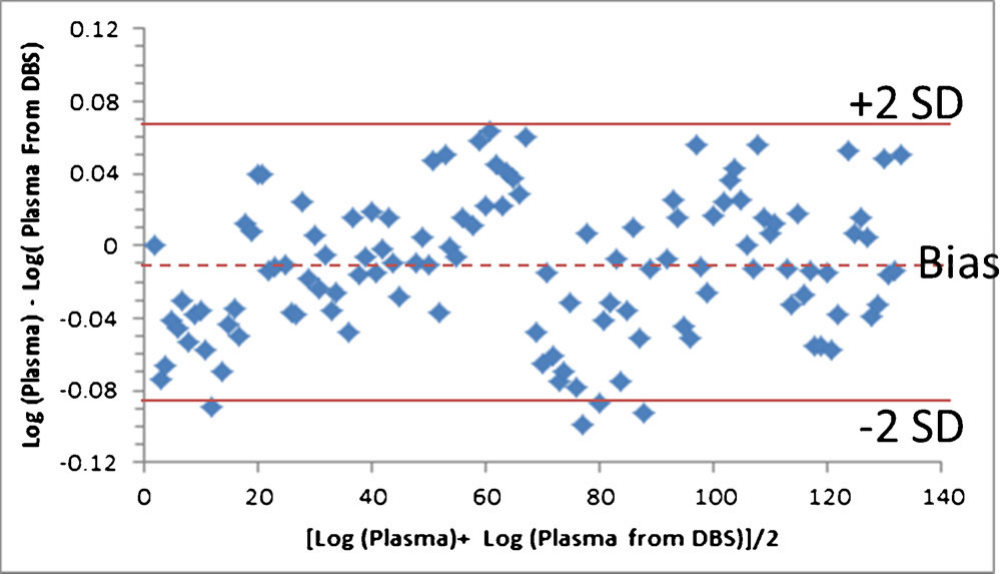
Bland–Altman plot comparing plasma and DBS concentrations for pharmacokinetic samples from the phase 1 study for MK-8931

For MK-8931, venous blood was drawn into a single EDTA collection vial and a small quantity spotted onto a DBS card; the remaining blood sample was centrifuged to extract plasma. MK-8931 concentrations were analyzed in both the DBS and the plasma samples. The DBS analytical method was based on either direct extraction or direct extraction followed by liquid–liquid extraction of MK-8931 from human dried blood spots on DMPK-A cards. The analyte from DBS samples and its stable isotope labeled internal standard contained in the extraction solvent were analyzed by HPLC-MS/MS. The lower limit of quantitation (LLOQ) was 1 ng/mL with a 3-mm punch size and was deemed adequate for clinical trials. Hematocrit was shown not to have an analytically relevant effect on MK-8931 concentration measurements in the range of 19.5 to 86.0%. Following the receipt of clinical study samples, incurred sample re-analysis (ISR) was completed to further validate the reproducibility of the assay methodology. Assay validation and clinical performance are summarized in Tables I and II. Bland-Altman plots comparing DBS and plasma concentrations showed a lack of bias between the methods (Fig. 2). The DBS analytical method was considered to have robust performance for use in a clinical setting.

**Table I:** Assay Validation Performance Summary for MK-8931

Assessment	Samples/Conditions Assessed	*N*	Mean Accuracy (%)	Mean Precision (Coefficient of Variation %)
Regression model analysis	Replicate standard curves (linear, 1 / x^2^)	5	92.0–105	3.7–7.3
Intra-run accuracy and precision at the LLOQ	3 core runs	5 in each run	101–111	9.2–18.9
Intra-run accuracy and precision at low, mid, and high QC	3 core runs	5 in each run	99.4–109	2.4–7.3
Inter-run accuracy and precision at LLOQ, low, mid, and high QC	Mean of 3 core runs in 3 days	3	101–107	0.8–4.9

**Table II:** Assay Clinical Study Performance Summary for MK-8931

Clinical Protocol	Mean Accuracy (%)				Mean Precision (Coefficient of Variation %)			
	*N*	QC L	QC M	QC H	*N*	QC L	QC M	QC H
PN0A	6	93.7	103	95.9	6	14.6	5.80	3.84
PN0B	40	99.9	102	101	40	8.44	2.87	5.21

In other instances, bioanalytical evaluations have ruled out DBS as a possible matrix. For example, one compound exhibited high levels of a circulating glucuronide metabolite and stability testing of DBS samples containing the glucuronide showed that it back-converted to parent compound at both room temperature and low temperature with acidification. This would confound the accurate measurement of the parent compound. Therefore, traditional plasma sampling with acidification to stabilize the metabolite was considered a more viable option, and DBS was not further pursued. In other instances, DBS has been successfully evaluated in early clinical studies, but the program failed to advance, and therefore, no further DBS data were collected (13).

### *In Vivo* Bridging

#### General Considerations

As most clinical development programs are initiated with, and likely to retain phase 1 studies with plasma assays, a quantitative *in vivo* relationship between blood and plasma concentrations is established through “bridging studies.” These typically encompass a staged evaluation commencing in a healthy subject study and then proceeding to a patient study. In each study, DBS and plasma samples are taken concurrently at various PK sampling time points that span a therapeutically relevant concentration range. Depending on whether the proposed future clinical trial (after bridging is established) would continue blood collection through venipuncture or in an out-patient setting, DBS may be collected *via* venipuncture in the clinic or through finger sticks. As sample collection has a direct impact on the quality of DBS assessments, Merck has developed extensive training material (including videos and educational materials) for sites for guidance on sample handling, and shipping. The participation of PKPD and/or BA scientists in start-up meetings is encouraged to ensure participating sites are appropriately educated on practical considerations of sample collection and handling.

For the healthy subject evaluation, an existing clinical pharmacology study (*e.g*., multiple ascending dose, control arm of a DDI, or special population study) should be leveraged. No *a priori* sample size is recommended; a typically sized clinical pharmacology study (*e.g*., 12–16 subjects) is sufficient. Subsequently, bridging data should be collected from an early patient study (*e.g*., phase 1b or POC).

Merck utilizes a “weight of evidence” approach that includes a series of graphical/descriptive evaluations and with equal or greater weightage given to population pharmacokinetic analyses. These analyses should follow a learn and confirm paradigm. Analyses should commence with the healthy subject study in an exploratory or “learning” mode and be confirmed in the patient study. Based on input provided for MK-8931 during the EMA oral hearing, an external qualification of the blood–plasma relationship is highly desirable. Establishing and qualifying this relationship enables pharmacometric analyses of concentration data across studies in a clinical program in support of the submission package and labeling.

#### Healthy Study

**Fig. 3:**
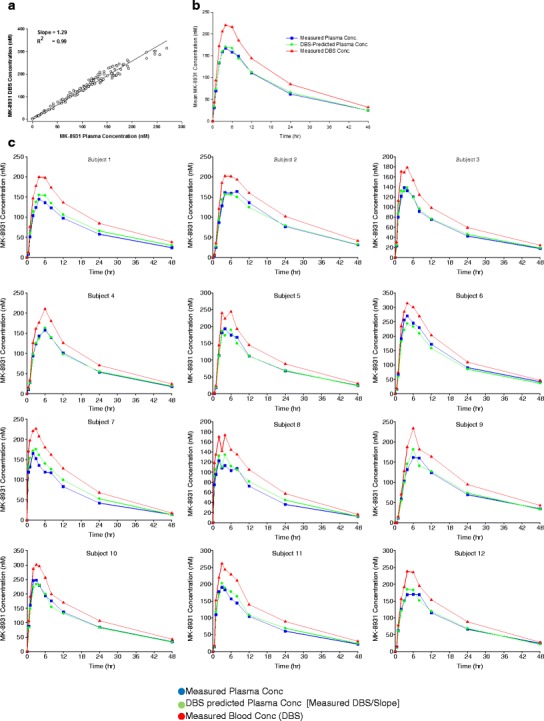
**a** Correlation of blood and plasma concentration data from a phase 1 bridging study of MK-8931. **b** Mean plasma and blood concentration-time data from a phase 1 bridging study of MK-8931. Note: DBS-predicted plasma concentrations were calculated as measured DBS divided by 1.29, the slope of the DBS-plasma linear regression line. **c** Plasma and blood concentration-time data for individual subjects from a phase 1 bridging study of MK-8931. DBS-predicted plasma concentrations were calculated as measured DBS divided by 1.29, the slope of the DBS-plasma linear regression line

For MK-8931, DBS was initially included along with plasma concentrations in healthy subjects in a clinical pharmacology study (12 subjects contributing 11 samples each). DBS samples were collected *via* venipuncture in the clinic. A number of graphical and descriptive analyses were conducted. Concurrently drawn DBS and plasma concentrations were plotted to explore data trends (Fig. 3a). The blood to plasma slope (95% CI) estimated by regression was 1.29 (1.27, 1.31), which was in close agreement with the *in vitro* estimated blood to plasma (B:P) ratio of 1.22. Of note, while regression fits may be applied to quantify the relationship, a prospective *R*^2^ cutoff should not be applied as a go/no go criterion. Further, non-linear trends should not be inferred as a lack of utility of DBS. Observed mean (Fig. 3b) and individual (Fig. 3c) concentrations were plotted by nominal time. The plots include MK-8931 concentration data derived from plasma, DBS, and “DBS-predicted plasma concentrations” (DBS concentration divided by the slope from regression fit of 1.29). The measured and DBS converted plasma concentrations were generally comparable and followed similar trends over time. These graphical and descriptive analyses suggested that the *in vivo* B:P relationship was well characterized in healthy subjects.

In Merck’s experience, the aforementioned graphical plots have been well received by regulators. Additionally, a mixed-effects modeling approach could be applied to evaluate the blood and plasma concentration data. In this approach, time-matched DBS and plasma concentration data pairs are fitted to a mixed-effects model (*e.g*., ln(DBS) = slope * ln (plasma)) in a program such as NONMEM. Inter- and intra-individual variability terms as well as the influence of covariates such as hematocrit may be evaluated using this approach.

**Fig. 4:**
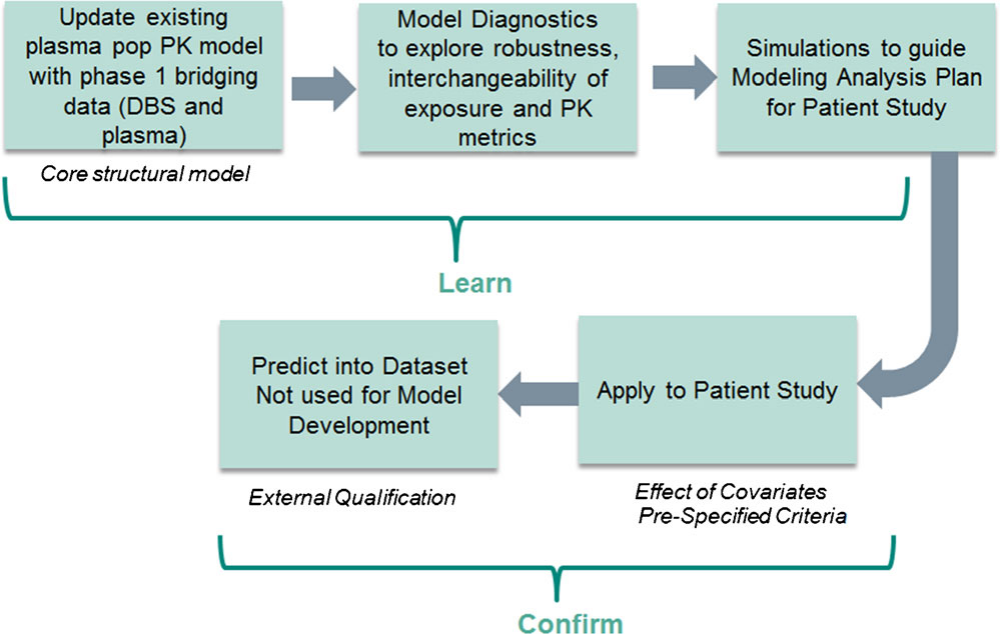
Road map for application of population PK to establish a quantitative bridge between plasma and DBS concentrations

**Fig. 5:**
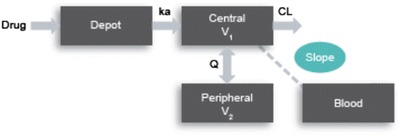
A base population pharmacokinetic model structure that relates plasma and DBS concentration data by a population estimated slope. See Appendix for example NONMEM code

Equal or greater weightage was applied to population PK as a critical element of the overall bridging strategy. Implementation followed a learn and confirm paradigm as shown in Fig. 4. MK-8931, DBS, and plasma concentration data from the phase 1 study were used to update an existing plasma population pharmacokinetic model. Blood concentrations were modeled as a separate compartment with an estimated population “slope” that related blood and plasma concentrations (Fig. 5). Separate residual error terms were applied for blood and plasma. Parameter estimates and errors were similar between a model that included plasma data alone and one that included plasma and DBS and were consistent with historical knowledge of the compound (Table III). The population slope (% relative standard error) estimated from the model was 1.27 (4.61%) and similar to that obtained from the regression analyses and the *in vitro* blood to plasma ratio. The impact of inter-individual variability on slope was explored and found not to be significant. Existing covariate relationships from the plasma model were applied to the plasma-DBS model; however, the impact of covariates on the slope was not evaluated at this stage. Random effects and residual errors for the parameters were within reasonable limits. Other key modeling parameters (*e.g*., clearance) were also well estimated which would be of relevance to support covariate analyses (a key objective for the phase 2/3 population PK evaluations).

**Table III:** Phase 1 MK-8931 Population PK Model Parameter Estimates (% Residual Standard Error) for Relevant Parameters

Parameter	Parameter Description	Plasma-Only Model^*a*^	Plasma + DBS Model^*b*^
		Estimate (%RSE)	Estimate (%RSE)
Slope	DBS/plasma ratio	–	1.27 (4.61)
σ^2^plasma	Additive residual variability for plasma	0.142 (7)	0.144 (7.29)
σ^2^DBS	Additive residual variability for DBS	–	0.186 (34.7)

^*a*^Model developed using phase 1 plasma data

^*b*^Model developed using Phase 1 plasma data as well as DBS data from a healthy volunteer bridging study

**Fig. 6:**
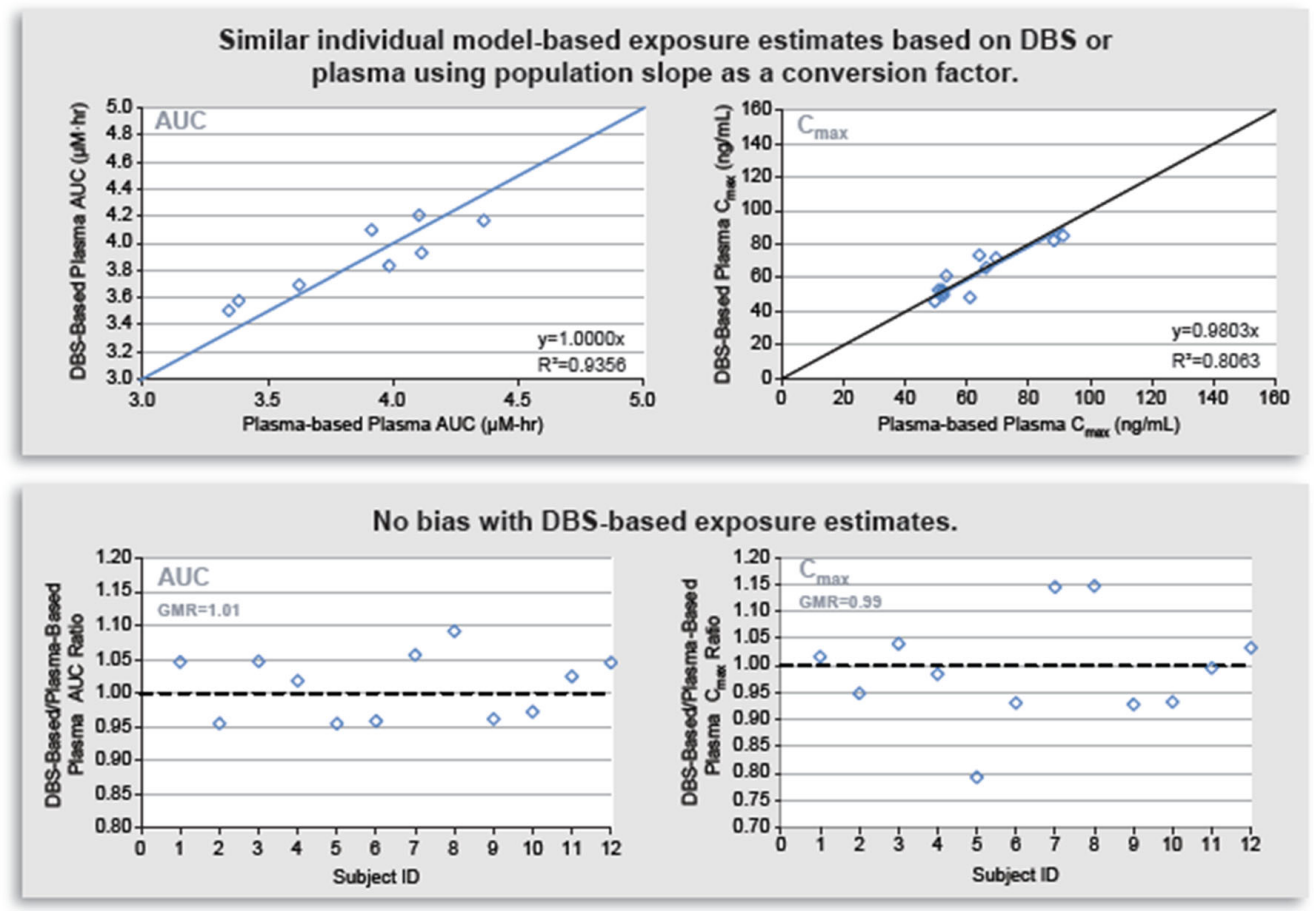
Individual MK-8931 model-predicted exposures using plasma alone data and model *vs.* from DBS concentration data converted to plasma using the model-estimated population slope

Modeling best practices and diagnostics were applied to explore the goodness of fit and the quantitative interchangeability of exposure metrics derived from the two matrices. Standard diagnostics and goodness of the fit plots such as visual predictive checks or CWRES (conditional weighted residuals) or IWRES (individual weighted residuals) *vs.* time plots differentiated by matrix were used to rule out the patterns of systematic bias or model mis-specification. Another useful diagnostic was to compare the individual post hoc plasma exposures from the plasma-DBS bridging study using (1) the plasma data from the bridging study and the plasma alone model and (2) the DBS data from the bridging study and the plasma + DBS model, using model-estimated slope to convert to a plasma exposure. DBS-based exposure estimates were similar and did not show any over- or under-prediction bias compared to the plasma-based estimates (Fig. 6).

Of note, while the blood to plasma relationship of MK-8931 was linear, if the underlying relationship for a given compound is non-linear, a more complex parametric description could be developed. However, the increased parameterization would need to be balanced relative to the overall advantages conferred by DBS to the program.

#### Patient Study

Descriptive and population analyses in healthy subjects supported the continued utility of DBS. Therefore a subsequent bridging dataset was developed in patients to confirm this relationship in a more heterogeneous and relevant patient population. For MK-8931, this was obtained from an early cohort of patients in a late-phase clinical trial. Concurrent DBS and plasma samples by venipuncture in clinic were taken pre-dose and as three sparse PK samples over a 13-week period. Additional DBS and plasma samples continued to be collected from the study based on advice received at an EMA oral hearing to develop an external qualification dataset.

**Fig. 7:**
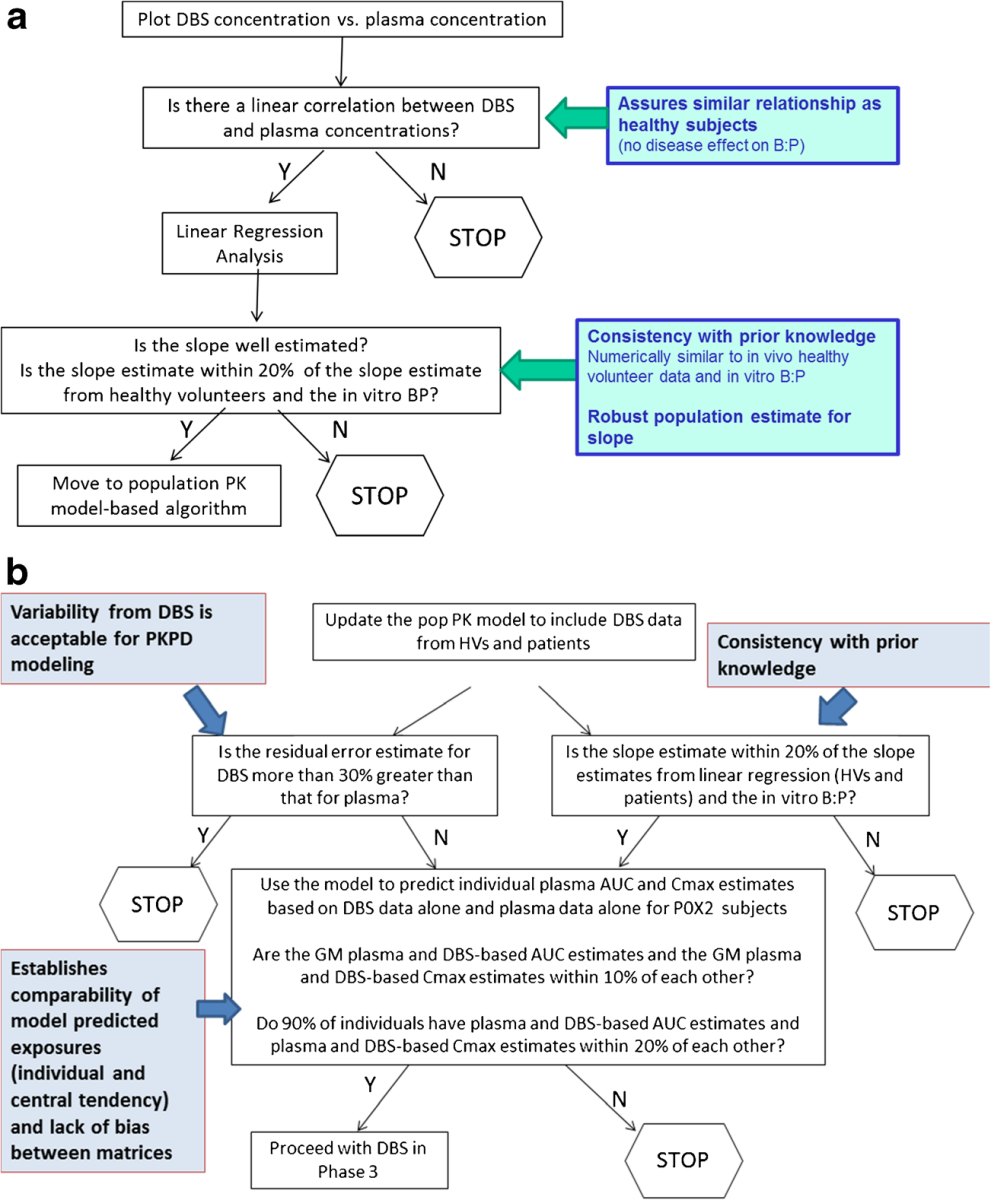
**a** MK-8931 DBS-plasma decision tree no. 1 (linear regression analysis based on patient data only). **b** MK-8931 DBS-plasma decision tree no. 2 (population PK model-based analysis based on healthy volunteer and patient data)

Simulations using the phase 1 pop PK model were used to develop prospective go/no go decision criteria and included in the modeling analysis the plan for the patient study to support the decision of whether DBS could be used as the sole matrix for future phase 3 studies. The modeling analysis plan would evaluate (i) comparability of model-estimated slope to regression estimated (*in vivo*) and *in vitro* blood to plasma ratio, (ii) similarity and lack of bias in parameters and post hoc exposures derived from plasma and DBS converted to plasma, and (iii) similar central tendency of plasma predicted *vs.* DBS converted plasma predicted exposures. For MK-8931, two decision trees were included in the modeling analysis plan (Fig. 7). These decision trees represent program-specific criteria and need to be adjusted on a case-by-case basis. Based on discussions at the EMA oral hearing, the modeling plan was updated to include an external qualification step where the DBS-plasma population PK model (including the population slope term) would also be used to estimate exposures (based on DBS data alone) for an additional set of patients not utilized for the model development.

A similar set of descriptive and graphical analyses as those performed on data from healthy subjects was planned for data from the patients. Furthermore, the population PK model would be updated with patient-derived DBS data and assessed relative to the proposed modeling plan and decision trees. Additional patient data from this data, not included in model development, would be used for external qualification. In its totality, the modeling plan was designed to evaluate whether DBS could achieve the broader PK objectives of the clinical program, such as application for exposure-response modeling, with a similar degree of fidelity as plasma. These pharmacometric assessments (as well as the preceding strategic, *in vitro* and bioanalytical components) were a critical aspect of the package submitted to regulatory agencies for feedback on whether DBS would be suitable as the sole matrix for late-stage studies. For blinding considerations, results from the patient study are not included in the current dissemination. These will be the subject of a subsequent external communication upon unblinding of the study.

### Regulatory Input

As clinical application of DBS is still emerging, regulatory guidance on DBS is yet to be established. Thus, feedback from key regulatory agencies is recommended in a timely manner for each program. The briefing package should present an integrated assessment of *in vitro* data, bioanalytical feasibility assessments, and *in vivo* bridging evaluations. The timing of such correspondence is program specific and should enable subsequent finalization of late-stage clinical plans. We recommend that meeting requests specify input from pharmacometric and bioanalytical reviewers. The inclusion of external expert opinions as part of the submitted dossier may be considered. It is Merck’s experience that comprehensive data packages that included the elements mentioned above enabled more productive regulatory interactions.

This approach was used for MK-8931. The focus of the interactions was to gain regulatory concurrence that the submitted package supported the use of DBS (collected in the clinic *via* venipuncture) as the sole method of PK sampling in ongoing and future MK-8931 late-stage clinical trials. For MK-8931, FDA interactions occurred at an earlier time frame when the DBS-associated pharmacometric package was not as well developed. The submitted package primarily relied on *in vitro* and BA feasibility assessments and descriptive/graphical analyses of phase 1 clinical data along with plans for the collection of plasma and DBS bridging data in patients, and the agency agreed that our proposal appeared reasonable. During correspondence, the agency recommended the use of the individual concentration time profiles as denoted in Fig. 3c. A comprehensive background document (inclusive of pharmacometric evaluations) was submitted for scientific advice to the CHMP/EMA. The agency requested an oral hearing as they indicated that this was their first regulatory experience of DBS. The key input received were the following:

- Merck has presented a comprehensive approach consisting of bioanalytical feasibility considerations and *in vitro* studies followed by an *in vivo* bridging program in both healthy and patient populations. Overall, this approach was considered robust to support the use of DBS as the sole source of PK data for the remainder of the MK-8931 phase 3 program.
- While the broader strategy was endorsed, the agency cautioned that the implementation of DBS requires unique considerations which may not be readily translatable to other development programs.
- There was a strong focus on trying to understand the impact of inter-individual variability on the slope and to ensure that the blood to plasma relationship (*i.e*., slope) could be applied to describe populations not included in the modeling dataset. The agency recommended that external qualification of the slope be demonstrated to show its predictive value in a dataset not used for model development.
- Of note, while the agency acknowledged the potential future benefits of home sampling, they refrained from providing commentary as they considered it to be technology in the early stages of development.

As stated before, the dissemination of this regulatory interaction is intended to advance the field by sharing knowledge in an area with relatively little regulatory precedence or guidance and as such is not intended to reflect a broader regulatory position statement.

## DISCUSSION

The strategy for using DBS in clinical programs at Merck has been developed over several years and has been a non-linear process. Merck arrived at its current state by looking at DBS holistically in the context of individual development programs, avoiding a one-size-fits-all approach. Early efforts mainly focused on analytical aspects, and it was only after a whole program approach was adopted, which the broader utility of DBS has started to be realized. Merck’s strategy requires a prospective and multi-study approach to build the data sets to enable successful clinical implementation. As such, the alignment of all groups (analytical, pharmacokinetics, clinical, operations) involved is critical to the successful implementation of DBS. The input from regulatory agencies has been critical to the refinement of our strategy. The feedback on both the analytical and pharmacokinetic aspects demonstrated scientific insightfulness and curiosity, while embracing a forward-looking attitude. As with any new approach, Merck anticipates unexpected hurdles during the development and implementation stages. Learning from the large-scale implementation of DBS in these trials is likely to benefit the broader community and will be the subject of future disseminations.

### Future Perspective

Merck believes that the strategies described above will help transform DBS from a niche bioanalytical technology to a practical clinical strategy that can be leveraged for population-based PKPD models suitable for regulatory submissions. Presentations of such data packages to regulators should build regulatory confidence in acceptance of DBS as a mainstream matrix and presumably influence future regulatory guidance. Merck believes that investing in novel methodologies such as these is an essential part of the solution to addressing rising drug development costs, while still meeting regulatory agencies expectations to demonstrate a robust understanding of the PKPD relationship of new therapeutic agents and demands by payers to reduce costs.

## APPENDIX

Population Analyses of DBS and Plasma Concentration Data: Excerpt from NONMEM Code within $ERROR Block

Q1 = 0

IF (CPT.EQ.2) Q1 = 1

IF (CPT.EQ.2) IPRED = LOG(F)

Y1 = IPRED + EPS(1)

Q2 = 0

IF (CPT.EQ.3 .AND. F.GT.0) Q2 = 1

IF (CPT.EQ.3 .AND. F.GT.0) IPRED = LOG(F * SLOPE)

Y2 = IPRED + EPS(2)

Y = Q1 * Y1 + Q2 * Y2
